# The Preparation of an Ultrafine Copper Powder by the Hydrogen Reduction of an Ultrafine Copper Oxide Powder and Reduction Kinetics

**DOI:** 10.3390/ma17071613

**Published:** 2024-04-01

**Authors:** Shiwen Li, Jianming Pang, Wei Han, Lingen Luo, Xiaoyu Cheng, Zhimin Zhao, Chaoran Lv, Jue Liu

**Affiliations:** 1 China Iron & Steel Research Institute Group, Beijing 100081, China; lsw9102@163.com (S.L.); luca1985@126.com (L.L.); lvchaoran_cat@163.com (C.L.); 2 College of Quality and Technical Supervision, Hebei University, Baoding 071002, China; jueliu@hbu.edu.cn

**Keywords:** mechanical milling, ultrafine, copper powder, hydrogen reduction, kinetics

## Abstract

Ultrafine copper powders were prepared by the air-jet milling of copper oxide (CuO) powders and a subsequent hydrogen (H_2_) reduction. After milling, the particle size and grain size of CuO powders decreased, while the specific surface area and structural microstrain increased, thereby improving the reaction activity. In a pure H_2_ atmosphere, the process of CuO reduction was conducted in one step, and followed a pseudo-first-order kinetics model. The smaller CuO powders after milling exhibited higher reduction rates and lower activation energies compared with those without milling. Based on the unreacted shrinking core model, the reduction of CuO powders via H_2_ was controlled by the interface reaction at the early stage, whereas the latter was limited by the diffusion of H_2_ through the solid product layer. Additionally, the scanning electron microscopy (SEM) indicated that copper powders after H_2_ reduction presented a spherical-like shape, and the sintering and agglomeration between particles occurred after 300 °C, which led to a moderate increase in particle size. The preparing parameters (at 400 °C for 180 min) were preferred to obtain ultrafine copper powders with an average particle size in the range of 5.43–6.72 μm and an oxygen content of less than 0.2 wt.%.

## 1. Introduction

Particles in size range of 10^−9^–10^−5^ m are commonly defined as ultrafine powders, including nanoparticles (10^−9^–10^−7^ m) and microparticles (10^−9^–10^−5^ m), which have been widely used in various fields [1]. Ultrafine copper powders have attracted increasing attention due to their high activity, large surface area, excellent electrical conductivity, and wide applications as powder metallurgical materials, electrically conductive pastes, and catalysts [2,3,4,5]. Several methods have been developed for the preparation of ultrafine copper powder, such as electrolysis [6], the mechanochemical method [7], metal vapor synthesis [8], pyrolysis [9], chemical reduction [10,11], and so on. Among these methods, chemical reduction, including liquid and gas-phase reduction, is a promising method owing to its convenient route and controllable process parameters. The liquid-phase reduction methods for obtaining ultrafine copper powder are based on the reduction of copper ions by reducing agents in the solution [12]. However, this approach is difficult to practically use because of its high cost, poor reaction ability, toxic and dangerous reductants (e.g., hydrazine and methanal), and the great deal of wastewater it involves [13,14]. The gas-phase reduction method utilizes reducing gases (e.g., H_2_, CO, or cracked natural gas) to reduce copper oxides to copper powders [15]. The traditional preparation method adopts an atomizing-oxidization-reduction technical route, in which bulk copper is first melted and broken into particles by a stream of gas or liquid, and then these particles are oxidized and ground to controllable particle sizes, followed by gas reduction and final treatments (milling and classification) to obtain the desired specifications [16]. In general, this technology is difficult to use to produce ultrafine copper powders on a large scale due to the fact that conventional atomizing and grinding methods hardly obtain a large number of ultrafine atomized copper or ultrafine copper oxide powders [17,18]. Nevertheless, most interesting of all, this route is facile and requires no chemical reagents compared to the aforementioned methods. Given that H_2_ is selected as the reducing agent, it would be a green and efficient production process (H_2_ + Cu_x_O → Cu + H_2_O) [19]. Therefore, if Cu_x_O powders are further crushed to the nano-scale or micron-scale level, followed by H_2_ reduction, ultrafine copper powder can be obtained.

It is well known that mechanical milling is a process in which mechanical forces are applied to a solid substance to induce the physicochemical changes, which has become a powerful tool for the preparation of new materials [20,21,22]. Owing to the advances in powder technology, ultrafine powders can be easily obtained by high-energy ball milling or jet milling [23]. Numerous studies have claimed that macroscopic and microstructural parameters of the powders change during mechanical milling, resulting in the significant decrease in particle size and increases in specific surface energy, grain boundary energy, dislocation energy, and partial amorphization energy, which improves the reactivity of the powders [20,24,25]. For instance, mechanochemical activation increased the reactivity of iron oxides, resulting in the decreased apparent activation energy (*E*) for the reduction of iron oxides via H_2_ [26,27]. Additionally, mechanical milling can synthesize many metal nanoparticles, such as iron, nickel, cobalt, and copper nanoparticles with a size range of 10–50 nm [7,28,29]. Although the preparation of nano-powder using mechanical milling is generally regarded as inefficient and costly; a large number of micron-scale materials are generated during milling, which is very beneficial and practical for the mass production of micro-powders in the size range of 0.5–10 μm [30,31,32]. Therefore, fabricating ultrafine powders that are less than 10 μm will be a more realistic goal.

Herein, we reported a method to fabricate ultrafine copper powders by mechanical milling and H_2_ reduction with the advantages of having a simple operation, being economical, being environmental friendly and being suited for large-scale production. The specific purposes of the study are to (i) explore the evolution of physicochemical properties of CuO powders in the air-jet milling process, (ii) investigate the thermodynamics and kinetics of the reduction of CuO by H_2_, and (iii) probe the effect of processing parameters on the properties of ultrafine copper powder.

## 2. Materials and Methods

### 2.1. Materials

Copper oxide powders (denoted as CuO-1, ≤300 mesh, CuO > 99.5%) were purchased from Jinchuan Group Co., Ltd., Jinchang, Gansu, China. Hydrogen (H_2_, 99.999%) and nitrogen (N_2_, 99.999%) were obtained from Beijing Chengweixin Gas Technology Co., Ltd., Beijing, China.

### 2.2. Preparation of Ultrafine Copper Powders

**Figure 1:**
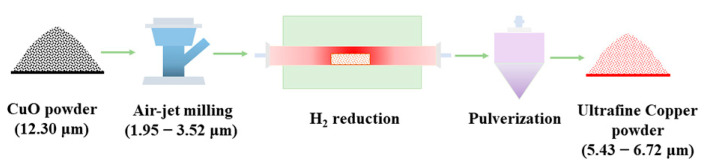
The schematic illustration for the preparation of ultrafine copper powders.

A two-step process was developed for preparing ultrafine copper powders (Figure 1). In the first step, a fluidized-bed jet mill (AB03, Jinghua Powder, Jining, Shandong, China) was used to comminute CuO powders (CuO-1). The chamber shell was made of SS304 stainless steel, lined with corundum. The comminuting medium was clean compressed air with a constant pressure of 0.8 MPa. By setting the classifier speed to 3000 r/min and 4000 r/min, two kinds of ultrafine copper oxide powders were obtained, with a corresponding average particle sizes (laser particle size D_50_) of 3.52 μm and 1.95 μm, which were denoted as CuO-2 and CuO-3, respectively. In the next step, we weighed about 1 kg of the powders (CuO-1, CuO-2, and CuO-3) and laid them on a corundum boat with an initial layer thickness of roughly 1.5 cm, and then transferred them into a tube furnace for reduction at 400 °C for 60 min in a H_2_ atmosphere. After cooling to room temperature, the samples were pulverized and classified by a nitrogen-jet mill, namely, Cu-1, Cu-2, and Cu-3, respectively. In addition, a series of control experiments were performed at different temperatures (200, 250, 300, 350, 400, 450, 500, and 600 °C) for 60 min. For comparative studies, the samples were also obtained following the same procedures at 400 °C for the holding time of 5, 15, 30, 60, 120, 180, and 300 min, respectively.

### 2.3. Characterization

The crystal phases were determined using an X-ray diffractometer (XRD, Ultima IV, Rigaku, Tokyo, Japan) using Cu Ka radiation at 40 kV and 40 mA with a scanning rate of 5°/min at a step size of 0.02°. The morphologies were performed on a field emission scanning electron microscope (SEM, Sigma 300, Zeiss, Jena, Germany). The samples were dispersed on the carbon double-sided tape, and then observed at an acceleration voltage of 15 kV using the secondary electron model. N_2_ adsorption–desorption isotherms, measured on a surface area and porosity analyzer (ASAP 2460, Micromeritics, Norcross, GA, USA), were applied to determine the specific surface area (SSA) using the Brunauer–Emmett–Teller (BET) method. The particle-size distribution and average particle size were measured on a dry laser particle-size analyzer (Bettersize 2600E, BETTER, Liaoning, China) at the refractive index of 2.86. Oxygen (O) and hydrogen contents were quantified by inert gas fusion analysis on an oxygen analyzer (O5500, NCS, Beijing, China) and a hydrogen analyzer (H5500, NCS, Beijing, China), respectively. The apparent density (*ρ*_a_) was determined using the Scott volumeter method. Specifically, we poured the powders into a funnel with a diameter of 2.5 mm and let them flow directly into the measuring cup (*V* = 25 cm^3^) until the cup was completely filled, then weighed the powders in the cup and calculated the apparent density (*ρ*_a_ = *m*/*V* = *m*/25). Thermogravimetric and differential thermal analysis (TG-DTA, METTLER TOLEDO, Zurich, Switzerland) was carried out to study the reduction behavior of CuO under pure H_2_. Specifically, in the non-isothermal tests, 20 mg of sample was reduced in a crucible at a heating rate of 10 °C/min, while in the isothermal tests, the same weight of the sample was first heated to the desired temperature (200, 250, 300, 350, or 400 °C) in a N_2_ atmosphere, and H_2_ was used to replace the N_2_ to trigger the reduction reaction.

## 3. Results

### 3.1. Evolution of Physicochemical Properties of CuO Powders in the Air-Jet Milling Process

**Figure 2:**
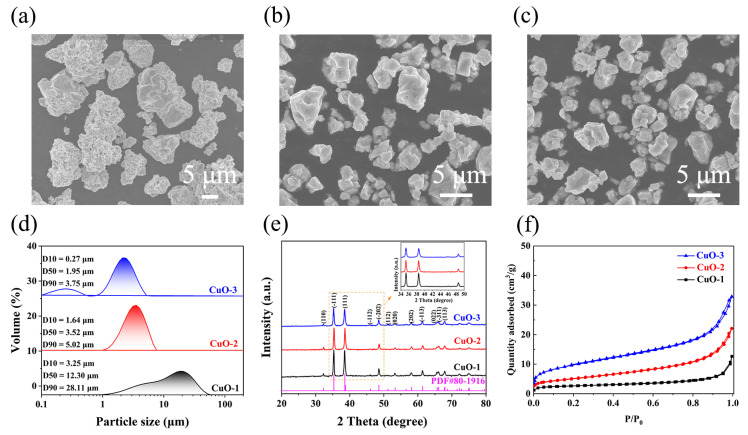
(**a**–**c**) SEM images, (**d**) laser particle-distribution curves, (**e**) XRD patterns, and (**f**) N_2_ adsorption–desorption isotherms of CuO-1, CuO-2, and CuO-3, respectively.

Figure 2a–c show the morphological evolution of CuO powders during the air-jet milling. Coarser CuO-1 particles presented irregular granules, which gradually evolved into fine polygonal particles (CuO-2 and CuO-3) after the milling treatment. The particle size ranges were 2.0–20.0 μm, 0.5–5.5 μm, and 0.2–4.5 μm for CuO-1, CuO-2, and CuO-3, respectively. The corresponding average particle sizes were further characterized using a laser particle-size analyzer based on the Mie theory (Figure 2d) [33]. Specifically, D_50_ corresponds to the particle size when the cumulative distribution reaches 50%, which is commonly used to indicate the average particle size of the powder. As seen from Figure 2d, the particle-size distribution of CuO powders narrowed after milling, and the D_50_ value of CuO-1 decreased from 12.30 μm to 3.52 μm (CuO-2) and 1.95 μm (CuO-3), showing that ultrafine CuO powders can be obtained by the air-jet milling process, which is a prerequisite for the preparation of ultrafine copper powders. 

The phase change during milling was analyzed using XRD (Figure 2e). The XRD patterns confirmed that all samples were composed of a single phase CuO (PDF#80-1916), demonstrating that no chemical reaction or phase change occurred during milling. Notably, compared with CuO-1, the diffraction peaks of activated CuO-2 and CuO-3 gradually broadened, and the peak intensities decreased, which might be attributed to grain refinement, dislocation multiplication, and partial amorphization during milling [34]. According to the Williamson–Hall equation (*γ*cos*θ* = *a*λ/*d* + 2*ε*sin*θ*, where *γ* is the integral breadth, *θ* is the Bragg angle, *a* is a constant value close to unity, *λ* is the wavelength, *d* is the average crystallite size, and *ε* is the structural microstrain), the average crystallite size and structural microstrain can be evaluated [35]. After milling, the average crystallite size of CuO powders decreased from 104 nm (CuO-1) to 78 nm (CuO-2) and 69 nm (CuO-3), whereas the structural microstrain increased from 0.03% (CuO-1) to 0.45% (CuO-2) and 0.71% (CuO-3) (Table 1). The BET-specific surface areas (*SSA*_BET_) were evaluated to be 0.51, 1.74, and 2.47 m^2^/g for CuO-1, CuO-2, and CuO-3, deduced from N_2_ adsorption–desorption isotherms, respectively (Figure 2f). Compared to CuO-1, the SSA_BET_ values of CuO-2 and CuO-3 increased by 3.41 times and 4.85 times, respectively, indicating the significant increase in the surface energy, which is conducive to CuO reduction [36]. In addition, the apparent density of the samples decreased from 3.47 g/cm^3^ (CuO-1) to 1.42 g/cm^3^ (CuO-2) and 1.16 g/cm^−3^ (CuO-3), reflecting that the voidage (*V*) of the powders was increased after activation (Table 1). According to the equation: *V* = 1 − *ρ*_a_/*ρ*_0_ (where *ρ*_0_ is the theoretical density of CuO, *ρ*_0_ = 6.51 g/cm^3^), the voidages were calculated to be 46.7%, 78.2%, and 82.2% for CuO-1, CuO-2, and CuO-3, respectively. That is, the voidage enhancement can result in the improved gas-permeability of the powders, which may be beneficial to H_2_ transfer and CuO reduction.

### 3.2. Thermodynamics of Reaction between CuO and H_2_

The thermodynamics of the reduction of CuO by H_2_ was studied. Previous studies have shown that the reaction between CuO and H_2_ proceeds in one or two steps [37,38], and the corresponding equations are as follows [39]:

One-step:

(1) $$\mathrm{CuO}\;\left(s\right)+{\;H}_{2}\;\left(g\right)=\;\mathrm{Cu}\;\left(s\right)+{\;H}_{2}O\;\left(g\right)\;\;\Delta_{r}G_{m}^{\theta }=-95,240-29.49\;T$$

**Figure 3:**
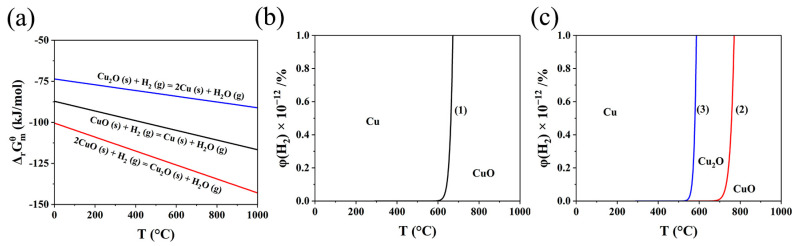
(**a**) $\Delta_{r}G_{m}^{\theta }$–T diagram for the reaction between CuxO and H_2_, (**b**) equilibrium diagram for one-step reduction of CuO by H_2_, and (**c**) equilibrium diagram for two-step reduction of CuO by H_2_.

Two-step:

(2) $$2\mathrm{CuO}\;\left(s\right)+{\;H}_{2}\;\left(g\right)={\;\mathrm{Cu}}_{2}O\;\left(s\right)+{\;H}_{2}O\;\left(g\right)\;\;\Delta_{r}G_{m}^{\theta }=-112,080-41.51\;T$$

(3) $${\mathrm{Cu}}_{2}O\;\left(s\right)+{\;H}_{2}\;\left(g\right)=2\mathrm{Cu}\;\left(s\right)+{\;H}_{2}O\;\left(g\right)\;\;\Delta_{r}G_{m}^{\theta }=-78,400-17.48\;T$$

where $\Delta_{r}G_{m}^{\theta }$ is the change in the Gibbs free energy of the reaction and *T* stands for Kelvin temperature. As shown in Figure 3a, the $\Delta_{r}G_{m}^{\theta }$ values of all the reactions, whether in one or two steps, are far less than zero, which demonstrates that the reaction between Cu_x_O and H_2_ occurs spontaneously and easily. Moreover, the equilibrium diagrams (Figure 3b,c) for reaction (1–3) can be plotted according to the following equations:

(4) $$K^{\theta }=\exp\left(-\frac{\Delta_{r}G_{m}^{\theta }}{RT}\right)$$

(5) $$\varphi_{H_{2}}=100/\left(1+K^{\theta }\right)$$

where $K^{\theta }$, *R*, and $\varphi_{H_{2}}$ represent standard equilibrium constant of the reaction, the gas constant, and the equilibrium composition of H_2_, respectively. Due to that $\Delta_{r}G_{m}^{\theta }$ << 0 and $K^{\theta }$ >> 1, $\varphi_{H_{2}}$ << 1%, which indicates that Cu_x_O can be reduced by trace amounts of H_2_ and that the corresponding reactions are irreversible.

### 3.3. Kinetics of Reaction between CuO and H_2_

**Figure 4:**
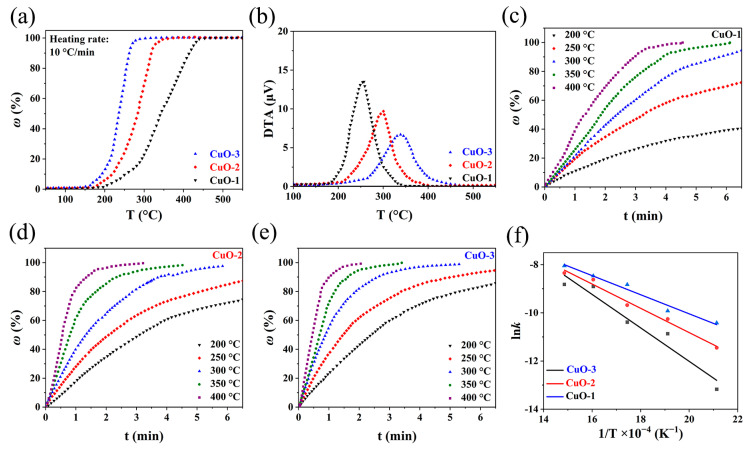
(**a**) Evolution of reduction extent of CuO powders with different particle sizes during the linearly programmed heating, (**b**) DTA curves, (**c**–**e**) the evolution of the reduction extent of CuO powders with different particle sizes at various temperatures, (**f**) and Arrhenius diagram for the reduction of CuO by H_2_.

The kinetics study of the reaction between CuO and H_2_ is of great significance, which not only offers deep insight into the reaction but also provides guidance for large-scale production. As mentioned earlier, in the reduction process, CuO is converted into metallic copper in a single step (CuO → Cu), or in sequential steps (CuO → Cu_2_O → Cu), but their behavior is different in nature. Figure 4a displays the reduction extent (% ω = wight loss × 100/20.12) of CuO powders with different particle sizes recorded during the linearly programmed heating. It is observed that the reduction extents of all samples were nearly 100%, indicative of the complete transformation of CuO to metallic copper. There was no hint of an intermediate phase on the curves except for the final transition platform, demonstrating that CuO was reduced to metallic copper in one step, which was consistent with the results reported in the previous literature [40,41]. In practice, kinetics conditions determine the formation of a suboxide during the process of CuO reduction. Under a normal supply of H_2_, CuO is reduced directly to metallic copper, whereas under small amounts of H_2_, the reduction of CuO follows a sequential pathway (CuO → Cu_2_O → Cu), which may be attributed to the fact that CuO can reach a metastable state that is less stable than Cu_2_O and react faster with H_2_ [41]. Furthermore, it was also found that the initial reaction temperature (about 185 °C, 160 °C, and 150 °C for CuO-1, CuO-2, and CuO-3, respectively) shifted leftwards, and the total reaction time (approximately 26 min, 16 min, and 12 min for CuO-1, CuO-2, and CuO-3, respectively) was shortened with decreasing CuO particle size, revealing that the smaller particle size lowered the reaction temperature and accelerated the reaction. Figure 4b presents rate functions for the reduction of CuO with different particle sizes. As can be seen, decreasing the particle size led to the significant enhancement of the reaction rate, with the order of CuO-3 > CuO-2 > CuO-1. Furthermore, the DTA curve can reflect the empirical order (*n*) of reaction. According to Kissinger’s method, the *n* values of CuO-1, CuO-2, and CuO-3 were calculated to be 1.01, 1.04, and 1.09 from the shape of the DTA peak, respectively, which verified that the reaction between CuO and H_2_ followed a pseudo-first-order reaction [42]. 

Figure 4c–e group the isotherms of CuO reduction with different particle sizes versus the reaction time at different temperatures. As can be observed, the reduction rate was accelerated and the reaction period was shortened with the decrease in the particle size, which was in line with the trend of the non-isothermal experiments. Meanwhile, the higher temperature likewise caused the increased reduction rate and reduction extent, suggesting that the apparent activation energy played a significant role. Bases on the above analysis, the reaction between CuO and H_2_ can be considered as a first-order reaction, and the rate of reaction is described by the following:

(6) $$-\frac{d\left(1-\omega \right)}{dt}=k\left(1-\omega \right)$$

Integration yields:

(7) $$k=\frac{\ln\left(1-\omega_{0}\right)-\ln\left(1-\omega \right)}{t}$$

where *t* is the time and *k* is the rate constant of reaction that can be deduced from the isotherm data. According to the Arrhenius law, the apparent activation energy of the reduction of CuO powders with different particle sizes was determined:

(8) $$\ln k=\ln A-\frac{E}{RT}$$

where *A* is the pre-exponential factor. Figure 4f depicts a linear relation between ln(*k*) and 1/*T*, where the slope reveals the apparent activation energy. The apparent activation energies for the reduction of CuO-1 to Cu by H_2_ were 57.44 kJ/mol, followed by 41.88 kJ/mol for CuO-2 and 32.96 kJ/mol for CuO-3 (Table 1). That is, reducing the particle size lowers the apparent activation energy, which is more conducive to CuO reduction.

**Figure 5:**
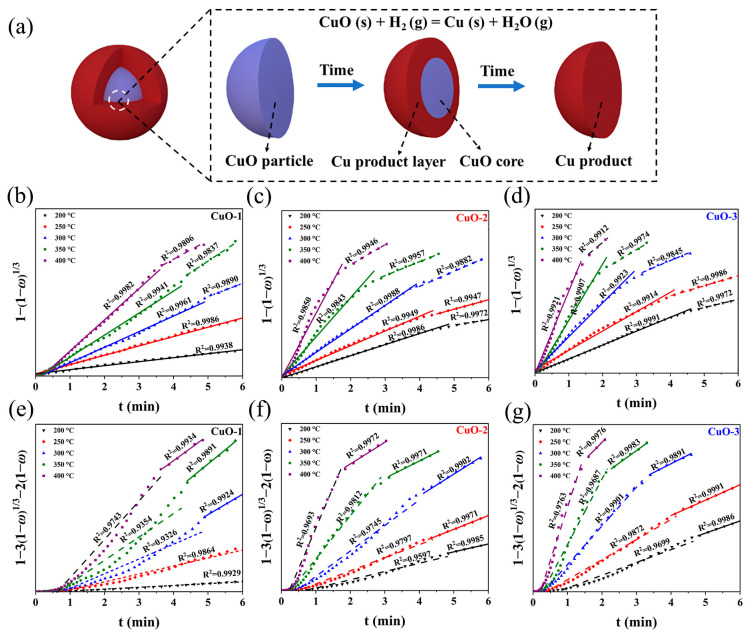
Kinetics analysis of CuO reduction via H_2_. (**a**) Schematics of unreacted shrinking core model, curve fitting of (**b**–**d**) interface chemical reaction and (**e**–**g**) diffusion through the product-layer-governing equations for CuO with different particle sizes at different temperatures.

Furthermore, it is also observed from the isotherms (Figure 4c–e) that the reduction extent of CuO first increased rapidly, and then slowed down with prolonged reaction time, suggesting that the whole reaction process might be controlled by multiple-stage reactions. According to the classical gas–solid reaction model, the reaction process between the gas and the solid oxide includes the following: (1) diffusion through a film covering the solid particle, (2) a chemical reaction at the interface, and (3) diffusion through a solid product layer [43]. In the process of the reduction of CuO via H_2_, the reaction initially occurs at the particle surface to form a solid copper product layer, and then the interface between the product layer and the unreacted core moves forward to the center of the particle as the reaction proceeds, which could be described by the unreacted shrinking core model (Figure 5a) [44]:

(9) t=ρr0c0−ce{ω3β+r06De[1−3(1−ω)23+2(1−ω)]+Kk′(1+K)[1−(1−ω)13]}

where *ρ* is molar density of CuO (mol/m^3^), *r*_0_ is particle radius, *c*_0_ is initial gas concentration, *c_e_* is gas equilibrium concentration, *β* is gas mass transfer coefficient, *D_e_* is effective diffusion coefficient of gas in the product layer, *K* is reaction equilibrium constant, and *k*′ is the rate constant of the interface reaction. 

If the reduction process is controlled by diffusion through a film covering the solid particle, Equation (9) can be simplified as the following [43]:

(10) $$t=\frac{\rho r_{0}\omega }{3\beta (c_{0}-c_{e})}$$

If the reduction process is limited by interface chemical reaction, Equation (9) can be written as the following [45]:

(11) t=ρr0c0−ce·Kμ(1+K)[1−(1−ω)13]

If the reduction process is dominated by diffusion through a solid product layer, Equation (9) can be represented as the following [46]:

(12) t=ρr026De(c0−ce)[1−3(1−ω)23+2(1−ω)]

That is, Equations (9)–(12) represent the contributions of diffusion through the film, the interfacial chemical reaction, and diffusion through the product layer, respectively. In this study, since the mass transfer of H_2_ is very fast, it can be assumed that the reaction process comprises the diffusion through the product layer and the interfacial reaction. Figure 5b–g depict that the dependence of 1−(1−ω)13 and 1−3(1−ω)23+2(1−ω) on reaction time (*t*). It can be found that the linear relationship between 1−(1−ω)13 and *t* is better than that between 1−3(1−ω)23+2(1−ω) and *t* in the first few minutes, suggesting that the interfacial reaction dominates the reduction of CuO in the initial-stage reaction. Whereas, the linear relationship between 1−3(1−ω)23+2(1−ω) and *t* is stronger than that between 1−(1−ω)13 and *t* in the later-stage reaction, indicating that the later stage of the reduction reaction is controlled by the diffusion through the product layer. Notably, temperature also affects the reaction between CuO and H_2_. The increase in temperature shortens the period of diffusion control in addition to reducing the total reaction time, which can be attributed to the fact that an elevated temperature is more beneficial to the transport of H_2_ in the product layer. Moreover, it is also observed that CuO with smaller sizes exhibit a shorter period of diffusion control during the reduction process, which is ascribed to the fact that that decreasing the particle size results in the formation of a thinner product layer and shorter diffusion distance. 

### 3.4. Effect of Technological Parameter on Properties of Ultrafine Copper Powders

The properties of the as-prepared copper powders may be practically affected by multiple factors, such as gas pressure, temperature, duration time, the thickness of the layer of CuO powders, the sintering between particles, etc. In general, temperature and duration time play a key role in the production of ultrafine copper powders. Although increasing the temperature and extending the holding time can effectively improve the reduction extent of the CuO powders, they also aggravate the sintering between the particles, thus resulting in the coarsening of the copper particles. In addition, the feeding amount will affect the reduction extent of the CuO powders, especially the oxygen content of copper powders. Therefore, the technological parameters were studied and optimized.

**Figure 6:**
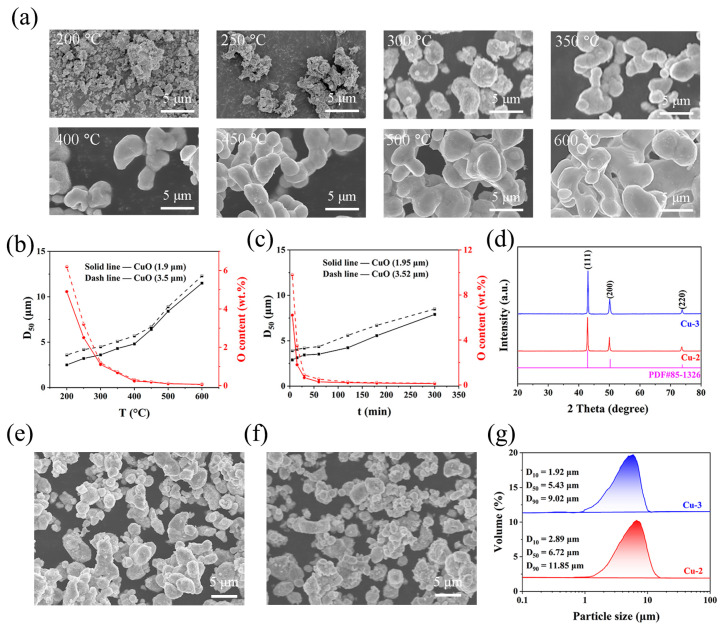
(**a**) Evolution of particle morphology with reduction temperature, (**b**) changes in D50 and oxygen content with temperature under the duration time of 60 min, (**c**) changes in D_50_ and O content with duration time at 400 °C, (**d**) XRD patterns of Cu-2 and Cu-3 (400 °C, duration of 180 min), SEM images of (**e**) Cu-2 and (**f**) Cu-3 (400 °C, duration of 180 min), and (**g**) laser particle-distribution curves of Cu-2 and Cu-3 (400 °C, duration of 180 min).

Figure 6a displays the evolution of particle morphology with reduction temperature. At temperatures lower than 250 °C, the product particles exhibited an irregular shape with cracks and pores at temperatures lower than 250 °C. These cracks and pores might be ascribed to the volume shrinkage stress resulting from the oxygen removal from CuO. There appeared to be little sintering between the particles. However, at temperatures higher than 300 °C, the surface of the product particles gradually became smooth, and sintering necks occurred between the particles. With increasing temperature, the sintering necks grew larger; especially, the size of the sintering necks almost approached that of individual particles when the temperature exceeded 500 °C. These results demonstrate that the sintering between the particles starts at 300 °C, which leads to the agglomeration and size increase in the particles. Figure 6b shows the effect of temperature on D_50_ and the oxygen content of the copper powders. With elevating the temperature from 200 °C to 600 °C, the oxygen content of both CuO-2 and CuO-3 samples first decreased rapidly, and then reached a platform (about 0.52 wt.%) after 400 °C. Meanwhile, the mean particle size gradually increased from 3.65 μm (200 °C) to 4.25 μm (400 °C) for CuO-2 and from 2.21 μm (200 °C) to 3.52 μm (400 °C) for CuO-3 below 400 °C, and jumped from 6.31 μm (450 °C) to 12.33 μm (600 °C) for CuO-2 and from 5.61 μm (450 °C) to 11.55 μm (600 °C) for CuO-3 exceeding 400 °C. Combining these results, 400 °C is a suitable temperature. The oxygen contents of both CuO-2 and CuO-3 samples sharply decreased to below 0.6% within 60 min, and then leveled off (Figure 6c). After a duration of 180 min at 400 °C, the oxygen contents of copper powder were 0.19 wt.% and 0.17 wt.% for CuO-2 and CuO-3, respectively. The effect of the duration time on particle size was relatively moderate. The mean particle size moderately increased from 3.91 μm (5 min) to 8.52 μm (300 min) for CuO-2 and from 2.89 μm (5 min) to 7.90 μm (300 min) for CuO-3 with increasing duration time (Figure 6b). Compared to the temperature, the duration time has less effect on the particle size, which might be ascribed to the fact that temperature exhibits a greater driving force on sintering than time. Therefore, combining these results, the production of ultrafine copper powders was preferred at 400 °C for 180 min.

In order to further probe the property of the as-prepared ultrafine copper powders, XRD, SEM, and a laser particle-size analyzer were used. The XRD patterns display that the sharp and strong peaks at 43.47°, 50.67°, and 74.68° were attributed to the (111), (200), and (220) planes of copper, confirming the formation of a pure-phase copper (PDF#85-1326) (Figure 6d). The SEM results show that the original irregular fine CuO particles were transformed into spherical-like copper particles through lattice oxygen removal and recrystallization (Figure 6e,f). It is also observed that a fine fraction aggregated to form a larger particle, which could be explained as the following: the atoms of small particles with high chemical potential spontaneously diffused to large particles with low chemical potential, resulting in the sintering between the particles with different sizes [47]. Cu-3 possessed a smaller particle size in a range of 1–8 μm compared with that of Cu-2 (3–10 μm). The laser particle-size analysis further confirmed the particle-size distribution of the as-prepared ultrafine copper powders. As shown in Figure 6g, Cu-2 and Cu-3 presented an average particle size of 6.72 μm and 5.43 μm, respectively. These results further demonstrate that reducing the particle size of CuO powders is conductive to obtain smaller copper powders. Additionally, the residual hydrogen content was evaluated. After hydrogen reduction, the hydrogen contents of Cu-2 and Cu-3 were measured to be 105 wt. ppm and 92 wt. ppm, respectively, indicating that the hydrogen was retained within the powders after reduction [48]. Altogether, using the technical route of the air-jet milling of CuO powders and hydrogen reduction, we can obtain ultrafine copper powders, which are expected to be applied in powder metallurgy, catalysts, electronic products, etc. [3,49,50].

## 4. Conclusions

A facile method of preparing ultrafine copper powder was developed, which involved the air-jet milling of CuO powders followed by H_2_ reduction. When CuO powders (D_50_: 12.30 μm) were ground to an average particle size of 1.95 μm, the specific surface area increased from 0.51 m^2^/g to 2.47 m^2^/g, the microstrain climbed from 0.03% to 0.71%, and the grain sized decreased from 104 nm to 69 nm. The thermogravimetric results demonstrated that decreasing the particle size of CuO powders accelerated the process of H_2_ reduction and lowered the apparent activation energy from 57.44 kJ/mol (D_50_: 12.30 μm) to 32.96 kJ/mol (D_50_: 1.95 μm). The reduction of CuO via pure H_2_ was a single-step reaction, which could be described by the pseudo-first-order model. The kinetics analysis indicated that the interface reaction dominated the initial stage of the reduction of CuO via H_2_, whereas the diffusion through the solid product layer controlled the later stage. Additionally, the effect of temperature on the oxygen content and particle size of copper powders was greater than duration time. The SEM results showed the sintering and agglomeration increased the particle size of copper powders. Optimally, ultrafine copper powders with an oxygen content below 0.2 wt.% and an average particle size range of 5.43–6.72 μm were fabricated at 400 °C for 180 min. This work provides some guidance for the production of ultrafine copper powder.

## Figures and Tables

**Table 1:** Physicochemical properties of CuO powders with different particle sizes and their apparent activation energies by H_2_ reduction.

Sample	D_50_ (μm)	*d* (nm)	*ε* (%)	*SSA_BET_* (m^2^/g)	*ρ*_a_ (g/cm^3^)	*E* (kJ/mol)
CuO-1	12.30	104	0.03	0.51	3.47	57.74
CuO-2	3.52	78	0.45	1.74	1.42	41.88
CuO-3	1.95	69	0.71	2.47	1.16	32.96

## Data Availability

The data that support the findings of this study are available on request from the corresponding author.
